# Potential role of N6-methyladenosine modification in the development of Parkinson’s disease

**DOI:** 10.3389/fcell.2023.1321995

**Published:** 2023-12-13

**Authors:** Jiale Zhou, Yang Han, Ruizhe Hou

**Affiliations:** ^1^ Key Laboratory of Zoonosis Research, Ministry of Education, College of Veterinary Medicine, Jilin University, Changchun, China; ^2^ Laboratory Animal Center, College of Animal Science, Jilin University, Changchun, China; ^3^ Department of Neurosurgery, China-Japan Union Hospital of Jilin University, Changchun, China

**Keywords:** N6-methylAdenosine (m6A), RNA methylation, methyltransferase, epigenetics, neurodevelopment, Neurological disease

## Abstract

N6-methyladenosine (m6A) represents the most abundant modification of messenger RNA (mRNA) and is regulated by methyltransferases (writers), demethylases (erasers), and m6A-binding proteins (readers). A dynamic modification process is implicated in nearly every critical stage of RNA metabolism, including mRNA stability, transcription, translation, splicing, nuclear export, and decay. Notably, m6A methylation is significantly enriched in the brain and has recently been shown to be associated with neurodevelopmental disorders and the development of Parkinson’s disease (PD). In this review, we summarize the proteins involved in the process of m6A modification and elucidate the emerging role of m6A modification in PD, which could illuminate alternative strategies for the prevention and treatment of PD.

## 1 Introduction

Epigenetics is a form of stable inheritance that does not change the basic sequence of the DNA and includes DNA methylation, histone modification, and RNA modification of both mRNA and non-coding RNA (ncRNA). Compared to DNA methylation and histone post-translational modification, RNA modification has been less thoroughly studied. However, the recent development of RNA sequencing technology has fostered increased research into RNA epigenetics, and more than 170 RNA modifications have been discovered (Wiener and Schwartz, 2021). These modifications are mainly m1A, m5C, m6A, m7G, *etc.* N6-methyladenosine (m6A) was initially identified in 1974. It is considered the most common internal transcriptional modification, especially in eukaryotic mRNA (Desrosiers et al., 1974; Dubin and Taylor, 1975; Fang et al., 2021).

The methylation of m6A describes the addition of a methyl group at the sixth nitrogen position of adenine and is recognized as a dynamic, reversible modification process (Jia et al., 2011). It is regulated by methyltransferases, demethylases, and m6A-binding proteins, called writers, erasers, and readers, respectively. M6A methylation occurs in various RNA species, including mRNA, tRNA, rRNA, small nuclear RNA, microRNA precursors, and long non-coding RNA (Punekar et al., 2013; Du et al., 2018; van Tran et al., 2019). The dynamic modification of m6A occurs in nearly all stages of RNA metabolism, including mRNA stability, transcription, translation, splicing, nuclear export, and decay (Alarcón et al., 2015a; Aguilo et al., 2015; Meyer et al., 2015; Yue et al., 2015; Du et al., 2016; Yoon et al., 2017; Zhao et al., 2017).

Parkinson’s disease (PD) is a chronic neurodegenerative disease that predominately affects the motor nervous system. Its key clinical symptoms include resting tremors, bradykinesia, myotonia, and postural imbalance. The pivotal pathological changes observed in PD are the degeneration and subsequent death of dopaminergic neurons in the substantia nigra (Lotankar et al., 2017).

M6A-specific methylated RNA immunoprecipitation (MeRIP) has revealed abundant m6A modifications in the brain (Meyer et al., 2012). As a result, an increasing number of studies have investigated the functional significance of m6A modification in the nervous system and its effects on normal physiology. In this review, we will summarize the enzyme proteins that contribute to the process of m6A modification and explore the emerging role and biological significance of m6A modification in PD. This will ultimately provide new insights into the diagnosis and treatment of PD.

## 2 M6A-related proteins

### 2.1 Writers

**FIGURE 1:**
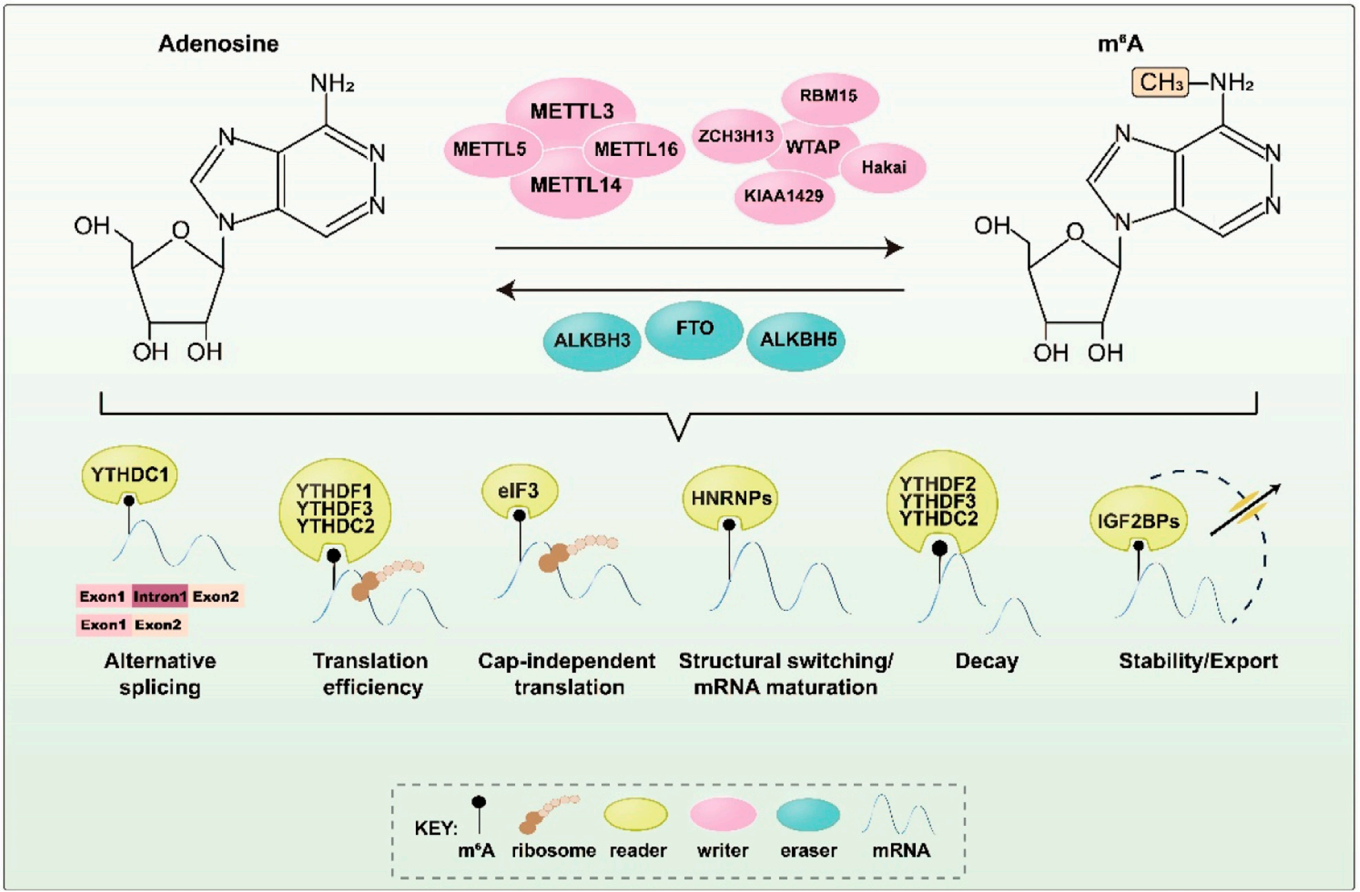
Key proteins and corresponding functions during m6A methylation. M6A methylation is regulated by m6A methyltransferase, demethylase, and m6A binding protein to perform a variety of biological functions. It affects critical stages of RNA metabolism and downstream gene expression regulation.

M6A methylation is mainly catalyzed by a methyltransferase complex (MTC), which includes METTL3, METTL14, WTAP, VIRMA/KIAA1429, RBM15, ZC3H13, and Hakai (Figure 1). METTL3, which is highly conserved in eukaryotes, was the first m6A methyltransferase to be discovered and is the most critical core component of the MTC, serving a catalytic function (Bokar et al., 1997; Geula et al., 2015). METTL14 principally acts as an RNA binding scaffold, stabilizing the MTC structure and enhancing METTL3’s catalytic activity. WTAP, a regulatory subunit of the RNA methyltransferase complex, links METTL3 to METTL14 and facilitates the dimer’s localization (Wang Y. et al., 2014; Liu et al., 2014; Ping et al., 2014). VIRMA/KIAA1429 recruits and guides the catalytic core methyltransferase components (METTL3/METTL14/WTAP) to specific RNA regions for m6A methylation (Yue et al., 2018). RBM15 and RBM15B, although they lack catalytic function, can bind to METTL3 and WTAP, directing these two proteins to specific RNA sites for m6A modification (Patil et al., 2016). ZC3H13 primarily promotes MTC’s binding to RNA, and its interaction with WTAP can substantially enhance the MTC’s catalytic function, regulating RNA m6A methylation in the nucleus (Wen et al., 2018). Although Hakai is less well-studied, it is also part of the m6A biogenesis mechanism in vertebrates and plants. In mammalian cells, Hakai strongly interacts with WTAP, and studies indicate that it is a core member of the m6A-modified protein family and an essential component of the MTC in *Drosophila* and human cells (Horiuchi et al., 2013; Bawankar et al., 2021).

In addition, METTL16 and METTL5 perform m6A methylation in a non-MTC-dependent manner. METTL6, a conserved U6 snRNA methyltransferase, controls SAM homeostasis by post-transcriptionally regulating the expression of SAM synthase genes (Pendleton et al., 2017). METTL5 is mainly responsible for catalyzing m6A modification on 18S rRNA (van Tran et al., 2019; Lei et al., 2023).

### 2.2 Erasers

The discovery of demethylases has proven that m6A methylation is a dynamic and reversible modification process. The currently recognized m6A demethylases are mainly FTO, ALKBH5, and ALKBH3.

FTO, also known as fat mass and obesity-associated protein, was the first m6A demethylase to be discovered and showed potential in regulating selective splicing and 3′ end mRNA processing and translation (Jia et al., 2011; Bartosovic et al., 2017; Zhang et al., 2019). FTO can catalyze the demethylation of both m6A and m6A.m., with preferences that are likely influenced by its subcellular localization (Mauer et al., 2017; Wei et al., 2018). In contrast, ALKBH5 (alkylation protein AlkB homolog 5), the second identified m6A demethylase, exhibits no activity toward m6A.m. substrates. It significantly influences mRNA output and RNA metabolism by reducing the level of m6A in nuclear speckles (Zheng et al., 2013). Both FTO and ALKBH5 catalyze the demethylation of m6A through an Fe(II)- and α-ketoglutarate-dependent mechanism, initially oxidizing m6A to N6-hydroxymethyladenosine (hm6A) before converting hm6A to N6-formyladenosine (f6A) and, eventually, transforming f6A into adenosine (A) to complete the demethylation process. Recent studies have reported that another AlkB family homolog, ALKBH3, also facilitates m6A demethylation in tRNA and enhances protein translation efficiency during cancer cell proliferation (Ueda et al., 2017).

### 2.3 Readers

The m6A reader mainly contributes to RNA recruitment. The YTH domain family, including YTHDF1, YTHDF2, YTHDF3, YTHDC1, and YTHDC2, are the first identified m6A readers. They bind directly to m6A through a common YTH domain (Dominissini et al., 2012). YTHDF1 promotes translation by elevating ribosome occupancy, recruiting translation initiation complex eukaryotic initiation factor 3 (eIF3), or working with YTHDF3 (Meyer et al., 2015; Wang et al., 2015; Shi et al., 2017; Lin et al., 2019). YTHDF2 contributes to mRNA stability and influences mRNA decay, while YTHDF3 affects the decay of methylated mRNA mediated by YTHDF2 (Wang X. et al., 2014; Shi et al., 2017; Fei et al., 2020). In the nucleus, YTHDC1 affects mRNA splicing and nuclear export, as well as mediates transcriptional repression by interacting with m6A-modified long non-coding RNA (lncRNA). Notably, YTHDC1 recognizes m6A-modified XIST to promote XIST-mediated X-chromosome silencing (Xu et al., 2014; Patil et al., 2016; Xiao et al., 2016; Roundtree et al., 2017). YTHDC2, which possesses 3'→5′ RNA helicase activity, selectively binds to m6A on its consensus motif and improves the translation efficiency of its target in spermatogenesis (Hsu et al., 2017; Wojtas et al., 2017).

In addition to the YTH domain family, heterogeneous nuclear ribonucleoproteins (HNRNPs), including HNRNPA2B1, HNRNPC, and HNRNPG, can also act as m6A readers. HNRNPA2B1 accelerates the processing of primary miRNA (pri-miRNA) by interacting with the DGCR8 protein in an m6A-dependent manner and regulates the alternative splicing of transcripts (Alarcón et al., 2015a; Alarcón et al., 2015b). Both HNRNPC and HNRNPG can modulate mRNA abundance and splicing after recognizing m6A (Zarnack et al., 2013; Liu et al., 2015; Liu et al., 2017). Moreover, insulin-like growth factor 2 mRNA-binding proteins (IGF2BPs), including IGF2BP1/2/3, enhance mRNA stability and translation efficiency in an m6A-dependent manner by recognizing the GG (m6A) C motif (Huang et al., 2018). FMRP, another m6A reader, can specifically bind to m6A-modified RNA and interact with CRM1 to promote the nuclear export of these RNAs, thereby regulating gene expression and influencing the development of neural stem cells and the nervous system (Edens et al., 2019).

## 3 Parkinson’s disease

### 3.1 Factors affecting the development of Parkinson’s disease

PD is a multifactorial neurodegenerative disease, the etiology of which has not been fully clarified to date, and no definitive and reliable clinical or testing tools currently exist to determine its cause. PD may be related to the interaction between age, environmental, and genetic factors.

Age is the greatest risk factor for developing PD. The prevalence of PD increases exponentially with age; 2%–3% of the population over 65 years of age is affected by PD (Pang et al., 2019; Kumar et al., 2022). In addition, 1-methyl-4-phenyl-1,2,3,6-tetrahydropyridine (MPTP), can induce typical Parkinson’s disease in both humans and primates. Certain environmental substances, including pesticides, herbicides, and specific industrial chemicals, share structural similarities with MPTP. Consequently, the environment could be an etiological factor in PD (Herrero et al., 1993; Nonnekes et al., 2018). Moreover, genetic factors significantly influence the development of PD, with data suggesting that genetic variation is found in 5%–10% of people with PD. In a comprehensive analysis using a large population-based twin registry, the heritability of PD for an age of diagnosis of less than 50 years was estimated at 0.83 (Goldman et al., 2019; Uwishema et al., 2022). Recently, genome-wide analyses of clinical cases of PD patients have identified a new set of PD-associated genes, including ANK2, DNAH1, and STAB1 (Yang et al., 2023).

### 3.2 Pathophysiological mechanisms of Parkinson’s disease

The two main hallmarks of PD pathophysiology are the accumulation of misfolded α-synuclein (α-Syn) and the decline of dopaminergic neurons in the substantia nigra (SN). α-Syn, a soluble protein that is predominantly found in the presynaptic and perinuclear regions of the central nervous system, is believed to be integral to cell membrane processes. Normal α-Syn exists as a single intrinsically disordered protein within the healthy brain (Fakhree et al., 2018). However, the misfolding and aggregation of α-Syn monomers results in the formation of pathological oligomers and protofibrils within neurons, which are associated with the progression of PD (Kalia et al., 2013).

Dopaminergic neurons are responsible for the storage and release of dopamine as a neurotransmitter and represent a widely distributed class of neurons within the brain that are crucially implicated in the regulation of significant physiological functions. The degeneration of nigrostriatal dopaminergic neurons is recognized as a central feature of PD, which is triggered by various factors, including mitochondrial dysfunction, Lewy body accumulation, neuroinflammation, excitotoxicity, and metal accumulation (Vallone et al., 2000).

## 4 M6A and Parkinson’s disease

### 4.1 Potential relationship between m6A erasers and PD

Chen et al. established cellular and rat models of PD using 6-hydroxydopamine (6-OHDA) and observed significantly reduced m6A levels in the striatal regions of the PD rat model and the PD cellular model. They found that ALKBH5 was notably elevated in the striatum of the PD brain, while FTO remained unchanged. Conversely, the PD cellular model exhibited upregulated FTO expression but no significant difference in ALKBH5. FTO knockdown increased m6A levels, subsequently inhibiting GRIN1 expression and reducing glutamate binding to receptors, thereby mitigating neurotoxicity (Chen et al., 2019).

Similarly, Geng et al. observed reduced total m6A levels and increased FTO expression in both an MPTP-treated PD mouse model and an MPP + induced-PD MN9D cell model. They suggested that FTO affects ATM expression by influencing the stability of ATM mRNA in dopaminergic neurons. FTO knockdown inhibited ATM expression, suppressing the upregulation of α-Syn and the downregulation of tyrosine hydroxylase (TH), thereby alleviating dopaminergic neuron death *in vitro* in a PD model (Geng et al., 2023).

Furthermore, FTO regulation appears to be closely linked to the control of neurotransmitter dopamine transmission (Hess et al., 2013). FTO inactivation may impair dopamine receptor type 2 (D2R) and type 3 (D3R)-dependent control of neuronal activity and behavioral response. FTO is thought to target GNAO1, GRIN1, and SYN1 to facilitate the D2R–D3R–G protein-coupled inward rectifier potassium signaling (GIRK) cascade, thereby promoting dopamine signal transduction through D2R and D3R signals. In addition, Qiu et al. identified five m6A-SNPs associated with PD, three of which were located in the ALKBH5 gene, suggesting that m6A-SNPs may contribute to the risk of PD (Qiu et al., 2020).

### 4.2 Potential relationship between writers, readers, and PD

He et al. compared m6A levels in peripheral blood mononuclear cells (PBMCs) from PD patients and healthy individuals. Their study revealed significantly reduced m6A levels in PD patients compared to healthy participants. The expression of m6a-related proteins, including METTL3, METTL14, and YTHDF2, was also notably downregulated. Further research indicated that METTL14 influences the stability of α-Syn mRNA and modulates α-Syn expression in an m6A-dependent manner, potentially offering valuable diagnostic information for PD (He et al., 2023).

Yu et al. established PD mice via MPTP and confirmed the aberrant expression of certain m6A-related proteins in the substantia nigra and striatum. They observed significantly reduced mRNA expression of RBM15b and YTHDF1 in the substantia nigra of PD mice, while IGF2BP1 expression was elevated. Moreover, in the striatum, the expression of RBM15, HNRNPG, METTL3, YTHDF1, HNRNPC, IGF2BP3, and RBM15 was markedly downregulated (Yu et al., 2022).

Furthermore, Gong et al. demonstrated that GLRX overexpression attenuated motor dysfunction and dopamine neuron degeneration in PD mice. They proposed that IGF2BP2 enhances GLRX mRNA stability in an m6A-dependent manner, whereas the knockdown of METTL3 substantially reduces the presence of GLRX mRNA enriched by an IGF2BP2-specific antibody. The study strongly associated m6A methylation modification with the progression of PD in mice (Gong et al., 2023).

Koranda et al. discovered that METTL14 deficiency leads to reduced m6A levels in the striatum without altering cell numbers or morphology. This deficiency also increased neuronal excitability and enhanced striatal sensitivity to dopamine agonist (DA) drugs, suggesting m6A’s essential role in maintaining striatal function and learning ability in adult mice (Koranda et al., 2018).

Additionally, Quan et al.’s data analysis suggested that HNRNPC might contribute to PD pathogenesis by inhibiting the proliferation of dopaminergic neurons, promoting their apoptosis, and inducing immune inflammation (Quan et al., 2021).

Overall, the emerging evidence indicates that m6A modifications play a pivotal role in the development of PD, presenting potential opportunities to prevent and treat this neurodegenerative disorder (Table 1).

**TABLE 1:** Potential relationship between m6A and Parkinson’s disease.

Related proteins	Explication	References
METTL3	Expression levels significantly reduced in patients with PD	He et al. (2023)
	Expression significantly downregulated in the striatum of PD mice	Yu et al. (2022)
	Influences the methylation level of GLRX mRNA and, thus, the IGF2BP2-GLRX pathway	Gong et al. (2023)
METTL14	Expression levels significantly downregulated in PD patients, affecting α-syn expression in an m6A-dependent manner	He et al. (2023)
	Deficiency increases neuronal excitability and striatal sensitivity to DA drugs	Koranda et al. (2018)
RBM15	Expression significantly downregulated in the striatum of PD mice	Yu et al. (2022)
RBM15B	Expression significantly reduced in the substantia nigra region of PD mice	Yu et al. (2022)
FTO	Suppresses GRIN1 expression and reduces glutamate binding to the receptor	Chen et al. (2019)
	Affects ATM expression through ATM mRNA stability, and FTO knockdown inhibits ATM expression	Geng et al. (2023)
	Inactivation may impair dopamine receptor type 2 (D2R) and type 3 (D3R)-dependent control of neuronal activity and behavioral responses	Hess et al. (2013)
ALKBH5	Presence of m6A-SNPs associated with PD	Qiu et al. (2020)
YTHDF1	Expression significantly downregulated in striatal and substantia nigra regions of PD mice	Yu et al. (2022)
YTHDF2	Expression levels significantly reduced in patients with PD	He et al. (2023)
HNRNPG	Expression significantly downregulated in the striatum of PD mice	Yu et al. (2022)
HNRNPC	Expression significantly downregulated in the striatum of PD mice	Yu et al. (2022)
	Inhibits proliferation and promotes apoptosis in dopaminergic neurons	Quan et al. (2021)
IGF2BP3	Expression significantly downregulated in the striatum of PD mice	Yu et al. (2022)
IGF2BP2	Enhances GLRX mRNA stability in an m6A-dependent manner	Gong et al. (2023)
IGF2BP1	Expression elevated in the substantia nigra region of PD mice	Yu et al. (2022)

## 5 Conclusion and prospects

As one of the most prevalent internal RNA modifications in eukaryotic mRNA, m6A methylation has emerged as a critical regulator of neuronal development and the pathogenesis of PD. This revelation offers a fresh perspective on the epigenetic regulation underlying Parkinson’s disease. Despite recent advancements, the significance of m6A modification in PD remains poorly understood, with several questions yet to be answered. For instance, the variations in m6A levels during different phases of PD development, the mechanisms responsible for these variations, and the correlations between m6A expression disparities in various tissue systems and the diversity of m6A functions remain largely unknown. Addressing these queries in future studies is crucial to unraveling the potential regulatory role of m6A in PD.

A comprehensive understanding of the role that m6A methylation plays in physiological homeostasis and disease, along with a deep exploration of the regulatory mechanisms governing the expression and function of m6A-associated proteins, is imperative. Components of these pathways may offer promising therapeutic targets for PD treatment. Delving into the intricacies of m6A modifications as PD progresses will also contribute to the investigation of PD-targeting therapies. The recognition of the critical role of m6A methylation in PD signifies a promising avenue for disease diagnosis and treatment, revealing new possibilities for future research.
